# Cannabis for pain: a cross-sectional survey of the patient information quality on the Internet

**DOI:** 10.1186/s42238-021-00093-x

**Published:** 2021-08-16

**Authors:** Jeremy Y. Ng, Darragh A. Dzisiak, Jessica B. Saini

**Affiliations:** Department of Health Research Methods, Evidence, and Impact, Faculty of Health Sciences, McMaster University, Michael G. DeGroote Centre for Learning and Discovery, Room 2112, 1280 Main Street West, Hamilton, Ontario L8S 4K1 Canada

**Keywords:** Cannabis, Marijuana, Pain, Quality of Information, Consumer Health Information, Internet

## Abstract

**Background:**

Cannabis has increasingly become an alternative treatment for chronic pain, however, there is evidence of concomitant negative health effects with its long-term usage. Patients contemplating cannabis use for pain relief commonly see information online but may not be able to identify trustworthy and accurate sources, therefore, it is imperative that healthcare practitioners play a role in assisting them in discerning the quality of information. The present study assesses the quality of web-based consumer health information available at the intersection of cannabis and pain.

**Methods:**

A cross-sectional quality assessment of website information was conducted. Three countries were searched on Google: Canada, the Netherlands, and the USA. The first 3 pages of generated websites were used in each of the 9 searches. Eligible websites contained cannabis consumer health information for pain treatment. Only English-language websites were included. Encyclopedias (i.e. Wikipedia), forums, academic journals, general news websites, major e-commerce websites, websites not publicly available, books, and video platforms were excluded. Information presented on eligible websites were assessed using the DISCERN instrument. The DISCERN instrument consists of three sections, the first focusing on the reliability of the publication, the second investigating individual aspects of the publication, and the third providing an overall averaged score.

**Results:**

Of 270 websites identified across searches, 216 were duplicates, and 18 were excluded based on eligibility criteria, resulting in 36 eligible websites. The average summed DISCERN score was 48.85 out of 75.00 (SD = 8.13), and the average overall score (question 16) was 3.10 out of 5.00 (SD = 0.62). These overall scores were calculated from combining the scores for questions 1 through 15 in the DISCERN instrument for each website. Websites selling cannabis products/services scored the lowest, while health portals scored the highest.

**Conclusion:**

These findings indicate that online cannabis consumer health information for the treatment/management of pain presents biases to readers. These biases included websites: (1) selectively citing studies that supported the benefits associated with cannabis use, while neglecting to mention those discussing its risks, and (2) promoting cannabis as “natural” with the implication that this equated to “safe”. Healthcare providers should be involved in the guidance of patients’ seeking and use of online information on this topic.

## Background

Pain is categorized into a number of different types, including acute (occurs suddenly and is usually associated with an injury), chronic (pain lasting for longer than 6 months, even after an injury has healed), nociceptive (pain stemming from the stimulation of pain receptors, usually in response to inflammation), and neuropathic pain (resulting from a dysfunction of the nervous system) (Santos-Longhurst 2018). According to a 2015 report, 17.6% of American adults experience severe levels of pain, and in 2016 an estimated 20.6% of Americans experience chronic pain (NIH analysis shows Americans are in pain 2018; Dahlhamer et al. 2018). Many of the individuals experiencing pain may consider or seek cannabis as a potential treatment option. While the long-term impacts of prolonged cannabis use remain understudied, there is preliminary evidence suggesting that negative health effects are concomitant with long-term usage, such as tuberculosis (with smoking cannabis), addiction (seen in 17% of heavy users who start using cannabis in adolescence), altered brain development, increased risk of schizophrenia, lowered IQ, and cyclic vomiting (Nugent et al. 2017; Schreiner and Dunn 2012; Volkow et al. 2014). Patients contemplating using cannabis for pain relief commonly seek information online but may not be able to identify trustworthy and accurate sources (Diviani et al. 2015). Therefore, it is important that healthcare providers are aware of the quality of such information commonly accessed by patients, in order that they are prepared to guide them in identifying trustworthy sources.

Few published studies have assessed the quality of online consumer health information specific to cannabis. One study examined the label accuracy of cannabidiol products sold online (Bonn-Miller et al. 2017), while others have evaluated the accuracy of cannabis claims found on popular websites (Sperry 2018), and information specific to cannabis addiction (Khazaal et al. 2008). Other studies have reviewed the quality of cannabis information published in magazines and newspapers (Halvorson et al. 2018; Montané et al. 2005). In general, the authors of these aforementioned studies concluded that the quality of cannabis information were often of very poor quality. Additionally, another study involved a qualitative analysis of online forum discussions on cannabis use and attention deficit hyperactivity disorder (ADHD) (Mitchell et al. 2016); the authors reported that the majority of information posted on these forums portrayed cannabis positively as a treatment option. They also found that online forum users believed that cannabis was encouraged by healthcare practitioners (Mitchell et al. 2016).

Approximately 4.5% of the internet searches worldwide are for health-related information, and a trend can be seen since 2004 that shows a steady increase in cannabis-related Google searches. In fact, from 2004 to 2016, cannabis-related searches have increased by 75% on Google.com (Morahan-Martin 2003; Lubin 2016). Despite this aforementioned published literature, to our knowledge, no research has assessed the quality of such information at the specific intersection of cannabis and pain, with the exception of a recently published study investigating the quality of online patient resources about cannabidiol for relief of hip or knee arthritis (Premkumar et al. 2021). Our study, however, is broader and applies to any type of pain in general. Given that a high prevalence of Americans experience severe pain (17.6%, among other pain types) it is important to evaluate the quality consumer health information available on this topic (NIH analysis shows Americans are in pain 2018). Therefore, the purpose of the present study is to assess the quality of cannabis consumer health information for the treatment/management of pain.

## Methods

### Search strategy and screening

A search strategy was developed to yield websites commonly visited by patients seeking information about cannabis for pain. Google was the only search engine used, as it holds 90%+ of the market share allowing us to replicate “typical” patient information-seeking behavior (Search Engine Market Share Worldwide 2020). Search terms were developed by JYN and included the following: “cannabis for pain”, “marijuana for pain”, and “weed for pain.” For each of the search terms, websites found on the first 3 Google search pages were considered for eligibility. We justified this decision based on the fact that past research has found that the first search page contains 92% of website traffic, with a 95% decrease for the second page, a 78% decrease for the third page, and subsequent decreases for each following page of results (Chitika Insights The value of Google result positioning 2013). Thus, searching beyond the third page would likely not reflect typical patient information-seeking behavior. DAD conducted the searches on 4 May 2020 across three countries that have either partially legalized or decriminalized cannabis as follows: Canada (Google.ca), the Netherlands (Google.nl), and the USA (Google.com). We searched Google across these three different countries, allowing our findings to be more generalizable and internationally representative, with respect to commonly visited websites. We specifically chose to search these three countries based on the fact that the use of medical cannabis has been legalized for approximately a decade in Canada, the Netherlands, and a number of states within the USA, allowing for the accumulation of a greater quantity of cannabis health-related information to exist online (University of Georgia 2020; Tattrie 2016; Centre For Public Impact (CPI) 2016). Searches were conducted using the Google Chrome browser in incognito mode to ensure that the websites retrieved were not influenced by previous browser search histories.

### Selection of quality assessment instrument

The DISCERN instrument is a questionnaire designed to assess the quality of written consumer health information. We selected the DISCERN instrument for the present study, as it has been found to be a valid and reliable tool for assessing the quality of publications (i.e., flyers, websites) about treatment choices. The DISCERN instrument consists of 16 questions, divided into three sections as follows: reliability of information (8 questions), specific details of information about treatment choices (7 questions), and an overall quality rating (1 question). Each item is rated based on a 5-point Likert scale, ranging from no/does not fulfill criterion (1 point) to yes/fulfills criterion (5 points) (DISCERN 2020; Charnock et al. 1999). It should be noted, however, that DISCERN cannot assess the validity of the written information, but rather the reliability of the information source (i.e., the DISCERN instrument cannot be used to judge the scientific accuracy of a publication’s sources).

### Eligibility criteria

DAD and JBS reviewed the search results from the first 20 websites on the first 3 Google webpages that were included for each search term, and duplicate websites across searches were removed. Websites were screened for eligibility and included if they contained at least one webpage that contained cannabis consumer health information for the treatment/management of pain. For the purpose of this study, we identified and included cannabis based on the definition provided by the World Health Organization: https://www.who.int/substance_abuse/facts/cannabis/en/. The following website types were excluded: general news websites (i.e., websites reporting on a wide range of topics and with no focus on cannabis or pain-specific information), peer-reviewed journals/articles, encyclopedia (i.e. Wikipedia) entries, non-English language websites, forums (i.e., Reddit), ebooks, major e-commerce websites (i.e., Amazon), video platforms (i.e., YouTube), websites targeted towards healthcare professionals rather than consumers, websites that were not publicly available, and websites that focused on cannabis as an addiction instead of as a treatment. While it is acknowledged that many of these websites may contain consumer health information (i.e., forums and videos), these were excluded because, as previously mentioned, the DISCERN instrument is designed for assessing written publications.

### Data extraction and website quality assessment

DAD and JBS data extracted the following items: website URL, website type, types of cannabis therapies, types of non-cannabis therapies (if present), whether the website appeared in more than one search (different search terms and/or regions), as well as scores for the sixteen DISCERN questions. For the purpose of this study, different webpages from the same website captured by searches were considered a single item for the purpose of DISCERN instrument quality assessment; we therefore conducted a quality assessment of websites and not individual webpages.

Following the identification of all eligible websites and to standardize the data extraction and the use of the DISCERN instrument, JYN, DAD, and JBS pilot tested its use on three separate websites and resolved any discrepancies across each item through discussion. The pilot test allowed for the standardization of how each DISCERN question is applied. Next, DAD and JBS independently completed the data extraction and assessed the quality of consumer health information on each eligible website using the DISCERN instrument. JYN then reviewed all scores with DAD and JBS to resolved any discrepancies that arose. The average of the two assessors’ scores was calculated for each question across all websites, providing an overall summed DISCERN score between 15 and 75, based on the scores for the first 15 questions. Additionally, the average scores and standard deviations for each DISCERN item was also calculated along with an average score for all 16 items.

## Results

### Search results

**Fig. 1:**
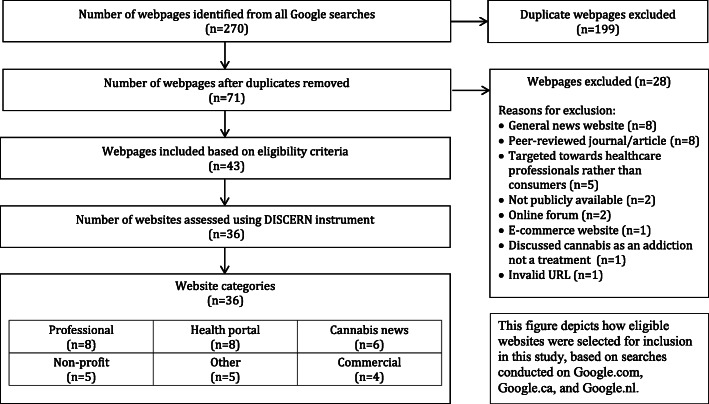
Web information search strategy and assessment flowchart

A total of 270 webpages were identified across searches, and after removing 199 duplicate webpages, 71 unique webpages remained. Twenty-eight webpages did not meet our exclusion criteria, for the following reasons: general news website (*n* = 8), peer-reviewed journal/article (n = 8), targeted towards healthcare professionals rather than consumers (*n* = 5), not publicly available (*n* = 2), online forums (*n* = 2), major e-commerce website (*n* = 1), discussed cannabis as an addiction not a treatment (*n* = 1), and invalid URL (*n* = 1). Of the remaining 43 webpages, 7 webpages belonged to websites already captured by the search and were collapsed into a single item. Therefore, 36 unique websites were deemed eligible for data extraction, and were assessed using the DISCERN instrument. This process is depicted in Fig. 1.

### General characteristics of eligible websites

Eligible websites were identified as belonging to 1 of 6 categories, as follows: health portal (websites that provide information on many types of diseases/conditions, *n* = 8), professional (websites marketing cannabis as a medical therapy, *n* = 8), cannabis news (websites that report specifically on emerging cannabis and pain related information, *n* = 6), non-profit (organization websites operating in a research and/or educational capacity, *n* = 5), commercial (websites that market cannabis products/services, *n* = 4), and finally, other (websites that do not fit into any of the aforementioned categories, *n* = 5). Of the 36 eligible websites, 28 appeared in multiple searches, while 8 appeared only once (3 websites from USA, 3 websites from the Netherlands, and 2 websites from Canada). Of the 36 websites, the following non-cannabis topics were discussed: surgery (*n* = 28), pharmaceutical medications (*n* = 28), and complementary and alternative medicine (CAM, *n* = 23). Full details associated with the general characteristics of eligible websites are shown in Table 1.

**Table 1:** General characteristics of eligible websites

Website name	URL	Website category	Cannabis therapies discussed	Non-cannabis therapies discussed	Appeared in more than one search?
Analytical Cannabis	https://www.analyticalcannabis.com	Cannabis News	Cannabis oils, edibles, vapes, and joints	NSAIDs, opioids	Yes
Arthritis	https://www.arthritis.ca	Non-profit	Cannabis creams, joints, edibles, vapes, and oils	Many surgical and pharmaceutical interventions listed for arthritis (medications, DMARDs), CAM therapies, and supplements (acupuncture, meditation, massage, and some herbs)	Yes
Beaver Bud	https://www.beaverbud.com	Other	Cannabis creams, vape, oils, joints, edibles, and capsules	None	No
CanaboMedical Clinic	https://www.canabomedicalclinic.com	Professional	Cannabis oils, joints, and edibles,	Discusses some pharmaceuticals; opioids, NSAIDs, and antidepressants, as well as exercise and physiotherapy, herbs and plants, meditation, massage, acupuncture, exercise, yoga	Yes
Canex	https://www.canex.co.uk	Cannabis news	Cannabis vape, oils, creams, edibles, and joints	Opioids, NSAIDS, and physiotherapy	Yes
Cannabis Clinics	https://www.cannabisclinics.ca	Professional	Cannabis vape and oils	None	Yes
Creaky Joints	https://www.creakyjoints.org	Non-profit	Cannabis vape, oils, scrubs, and edibles	Some pharmaceuticals, anti-inflammatory diet, some CAM therapies and supplements such as acupuncture, reiki, tai chi, and fish oil	Yes
Denver Dispensaries	https://www.denverdispensaries.net	Commercial	Cannabis joints, edibles, and vape	None	Yes
Doctor Oz	https://www.doctoroz.com	Other	Cannabis oils, joints, and edibles	Opioids, Botox, surgery, heat/cold therapy, some pharmaceuticals, CAM therapies and medicines; fish oil, fresh ginger, guided meditation, massage therapy, yoga, gua sha, hvla, helichrysum oil, herbal plaster	Yes
The Growth Op	https://www.thegrowthop.com	News	Cannabis vape, lube, oils, creams, edibles, and joints	Analgesics, NSAIDs, immunosuppressants, corticosteroids, counterirritants, and yoga	No
Guidance PA	https://www.guidancepa.com	Other	Cannabis vape, salves, oils, patches, joints, and edibles	None	No
Health Central	https://www.healthcentral.com	Health portal	Cannabis vape, oils, creams, edibles, lube, and joints	Surgery, various pharmaceuticals, hormone therapy, DMARDs, NSAIDs, and CAM therapies and supplements; acupuncture, the alexander technique, visceral manipulation, and Omega-3 fatty acids	No
Health Europa	https://www.healtheuropa.eu	Cannabis news	Cannabis tincture, oils, edibles, patches, balms, drinks, and capsules	Some surgery and pharmaceuticals, yoga, ginger	Yes
Health Harvard	https://www.health.harvard.edu	Professional	Cannabis vape, edibles, patches, capsules, and oils	TENS, surgery, physical therapy, and various pharmaceutical interventions, yoga, mind body therapies, acupuncture, massage therapy, tai chi, chiropractic, fish oil, hypnosis	Yes
Healthline	https://www.healthline.com	Health portal	Cannabis oils, edibles, joints, lotions, and gels	Medications, electrical stimulation, nerve block, surgery, and CAM therapies and CAM supplements; meditation, cloves acupuncture, yoga, biofeedback, turmeric, willow bark, essential oils, and ginger	Yes
Kevin MD	https://www.kevinmd.com	Health portal	Cannabis vape, oils, and edibles	Discusses conventional treatments such as surgery, pharmaceuticals, and physical therapy, acupuncture, and gingko biloba	Yes
Lab Blog UofM	https://labblog.uofmhealth.org	Professional	Cannabis vape and joints	Surgery, Psychological counseling, and pharmaceutical interventions, CAM therapies; tai chi, mindfulness, and herbal supplements	Yes
Leafly	https://www.leafly.ca	Commercial	Cannabis terpenes, edibles, joints, oils, vape, and drinks	Opioids	Yes
Marijuana Doctors	https://www.marijuanadoctors.com	Professional	Cannabis oils, joints, creams, edibles, vape, and patches	Electrical stimulation, medications, nerve blocks, surgery, and acupuncture	Yes
Med Card Now	https://www.medcardnow.com	Professional	Cannabis joints, oils, and creams	None	Yes
Medical Cannabis	https://www.medicalcannabis.com	Non-profit	Cannabis edibles, oils, and tinctures	Opioids	Yes
Medical News Today	https://www.medicalnewstoday.com	Health portal	Cannabis vape, oils, edibles, patches, and capsules	NSAIDs, opioids, nerve blocks, psychotherapy, TENS, surgery, heat/cold, and trigger point injections, and CAM therapies and supplements; such as deep tissue massage, acupuncture, meditation, Chinese herbal medicine, rosemary, turmeric, peppermint, eucalyptus and yoga	Yes
National Pain Report	http://www.nationalpainreport.com	Cannabis news	Cannabis vape, oils, capsules, sprays, creams, and edibles	Surgery and pharmaceutical interventions for pain (opioids), acupuncture, meditation, yoga, massage, and aromatherapy	Yes
Ohio Marijuana Card	https://www.ohiomarijuanacard.com	Professional	Cannabis terpenes, vape, joints, oils, capsules, and creams	None	Yes
People's Cali	https://www.peoplescali.com	Commercial	Cannabis vape, oil, edibles, drinks, tinctures, joints, and creams	Opioids	No
Practical Pain Management	https://www.practicalpainmanagement.com	Health portal	Cannabis terpenes, oils, edibles, vape, patches, and capsules	Interventional pain management; TENS, hormone therapy, anti-nerve growth factor meds, physical therapy, and CAM therapies and supplements; yoga, laser, magnets, flaxseed, and homeopathy	Yes
Psychology Today	https://www.psychologytoday.com	Health portal	Cannabis terpenes, vape, edibles, and oils	Surgery, pharmaceuticals, cognitive behavioral therapy, and TENS, mindfulness, biofeedback, ginger root and yoga	No
Rheumatoid Arthritis	https://www.rheumatoidarthritis.org	Non-profit	Cannabis oil	Discusses surgery exercise, diet, and a wide variety of medications (i.e. DMARDs, NSAIDs) in relation to arthritis treatment, briefly discusses mindfulness, massage, acupuncture, ginger and garlic	No
Safe Access Now	https://www.safeaccessnow.org	Non-profit	Cannabis oil, salve, vape, edibles, lotions, and balms	Opioids	Yes
SepaPain	https://www.sepapain.com	Professional	Cannabis (general—no specific therapies discussed)	Surgery and conventional pharmaceutical treatments; steroid injections, epidural, Botox, suboxone therapy, and stem cell therapy, yoga, and acupuncture	Yes
Very Well Health	https://www.verywellhealth.com	Health portal	Cannabis oil, capsules, joints, and vape	Surgery, pharmaceuticals, opioids, physical therapy, and antidepressants, massage therapy, acupuncture, and gingko biloba	Yes
Way of Leaf	https://www.wayofleaf.com	Other	Cannabis oil, clothing, face masks, drinks, edibles, and ointment	Some pharmaceuticals	Yes
WebMD	https://www.webmd.com	Health portal	Cannabis oil, edibles, and vape	Drug therapy, trigger point injections, surgical implants, TENS, bioelectric therapy, physical therapy, psychological treatment, nutritional supplements, and dietary approaches, yoga, biofeedback, Ginger, Gingko, meditation, and visualization	Yes
Weed Maps	https://www.weedmaps.com	Commercial	Cannabis gum, lube, vape, edibles, and drinks	None	No
Weed News	https://www.weednews.co	Other	Cannabis oil, edibles, joints, creams, vape, salves, and lube	None	Yes
WikiLeaf	https://www.wikileaf.com	Cannabis news	Cannabis terpenes, vape, oils, joints, edibles, and drinks	Some CAM remedies (pinene)	No

*Abbreviations*: *CAM* Complementary and alternative medicine, *DMARDS* Disease-modifying anti-rheumatic drugs, *NSAIDs* Non-steroidal anti-inflammatory drugs, *TENS* Transcutaneous electrical nerve stimulation

Table 1 provides detailed characteristics of each eligible website. Websites were deemed eligible and included if they contained at least one webpage that contained cannabis consumer health information for the treatment/management of pain

### DISCERN instrument ratings

The mean summed DISCERN score was 48.85 (SD = 8.13, range from 33.50 to 65.00). The mean overall score (question 16) was 3.10 (SD = 0.62, range from 2.00 to 4.50). The three highest scoring websites were Medical News Today (65.00), WebMD (64.50), and Very Well Health (62.50). The lowest scoring websites were SepaPain (37.50), People’s Cali (37.50), and Denver Dispensaries (33.50). DISCERN scores for each eligible website are provided in Table 2.

**Table 2:** DISCERN instrument ratings

Section		Section 1: Is the publication reliable?								Section 2: How good is the quality of information on treatment choices?							Section 3: Overall rating of the publication		
DISCERN Question		1. Are the aims clear?	2. Does it achieve its aims?	3. Is it relevant?	4. Is it clear what sources of information were used to compile the publication (other than the author or producer)?	5. Is it clear when the information used or reported in the publication was produced?	6. Is it balanced and unbiased?	7. Does it provide details of additional sources of support and information?	8. Does it refer to areas of uncertainty?	9. Does it describe how each treatment works?	10. Does it describe the benefits of each treatment?	11. Does it describe the risks of each treatment?	12. Does it describe what would happen if no treatment is used?	13. Does it describe how the treatment choices affect overall quality of life?	14. Is it clear that there may be more than one possible treatment choice?	15. Does it provide support for shared decision-making?	16. Based on the answers to all of the above questions, rate the overall quality of the publication as a source of information about treatment choices	Standard deviation of overall score (Q16)	DISCERN Score (sum of Q1-Q15)
Medical News Today	https://www.medicalnewstoday.com	4.50	4.50	4.50	4.50	5.00	5.00	5.00	4.50	4.00	4.50	4.00	2.50	3.00	4.50	5.0	4.50	0.71	65.00
WebMD	https://www.webmd.com	4.50	5.00	4.50	4.50	5.00	4.50	3.50	4.50	3.50	5.00	4.50	2.50	3.50	5.00	4.50	4.50	0.71	64.50
Very Well Health	https://www.verywellhealth.com	3.50	4.50	4.50	5.00	5.00	4.50	4.50	3.50	4.00	5.00	3.50	2.50	3.50	5.00	4.00	4.00	0.00	62.50
Creaky Joints	https://www.creakyjoints.org	4.50	4.50	4.50	4.00	1.50	4.50	4.50	4.50	3.50	4.00	4.50	2.50	4.00	5.00	4.50	4.00	0.00	60.50
Analytical Cannabis	https://www.analyticalcannabis.com	4.50	4.50	4.00	4.50	5.00	4.00	5.00	3.50	3.50	3.50	3.50	2.00	2.50	5.00	4.50	3.50	0.71	59.50
Healthline	https://www.healthline.com	4.50	4.50	4.00	4.50	5.00	4.00	5.00	3.50	3.50	3.50	3.50	2.00	2.50	5.00	4.50	3.50	0.71	59.50
Practical Pain Management	https://www.practicalpainmanagement.com	4.50	4.00	2.50	5.00	5.00	4.50	2.00	4.50	3.50	3.50	3.50	1.50	2.50	5.00	4.50	3.50	0.71	56.00
Health Central	https://www.healthcentral.com	4.50	4.50	4.50	4.50	3.50	3.50	4.50	3.00	2.50	4.50	3.00	2.00	2.00	4.50	4.00	3.50	0.71	55.00
Rheumatoid Arthritis	https://www.rheumatoidarthritis.org	4.50	5.00	2.50	3.50	5.00	2.50	4.50	2.50	3.00	4.00	3.50	3.50	2.00	4.50	4.50	3.50	0.71	55.00
Arthritis	https://www.arthritis.ca	4.00	3.50	3.50	2.00	2.00	3.50	3.50	4.00	3.00	4.00	4.50	4.00	2.50	5.00	4.50	4.00	0.00	53.50
Marijuana Doctors	https://www.marijuanadoctors.com	4.50	4.50	4.00	3.50	2.00	2.00	4.50	2.50	4.50	5.00	3.50	2.50	3.50	2.50	4.50	3.50	0.71	53.50
Health Harvard	https://www.health.harvard.edu	4.50	4.50	4.50	2.50	2.00	2.50	3.50	4.50	3.50	4.50	3.50	1.50	2.00	4.50	4.50	3.50	0.71	52.50
Kevin MD	https://www.kevinmd.com	3.50	3.50	2.50	2.50	2.50	3.50	4.50	4.50	3.00	4.00	4.50	2.00	2.50	4.50	3.50	3.50	0.71	51.00
Leafly	https://www.leafly.ca	4.00	3.50	3.50	4.00	3.50	3.50	4.50	3.00	4.50	4.50	3.50	1.00	2.50	1.50	3.00	3.00	0.00	50.00
Psychology Today	https://www.psychologytoday.com	2.50	2.00	2.50	3.50	4.00	4.50	3.50	4.00	4.00	4.00	3.50	2.50	2.00	4.50	2.50	3.00	0.00	49.50
Lab Blog UofM	https://labblog.uofmhealth.org	3.50	4.50	2.50	3.50	3.50	3.50	4.50	4.00	2.00	3.00	3.50	1.50	1.50	4.50	3.50	3.50	0.71	49.00
Canabo Medical Clinic	https://www.canabomedicalclinic.com	3.50	4.00	3.50	2.50	2.50	2.50	4.50	3.00	2.50	4.50	2.50	1.50	4.00	2.50	5.00	2.50	0.71	48.50
Beaver Bud	https://www.beaverbud.com	4.00	5.00	3.50	3.00	2.50	1.00	4.50	2.00	3.50	5.00	3.50	2.00	3.00	2.00	4.00	3.00	0.00	48.50
Way of Leaf	https://www.wayofleaf.com	4.50	3.50	3.50	3.50	3.50	2.50	4.50	3.00	3.50	4.50	3.00	1.00	2.50	1.50	3.50	3.00	0.00	48.00
The Growth Op	https://www.thegrowthop.com	4.50	4.50	3.50	3.00	2.50	2.50	4.50	3.50	3.50	4.50	2.00	1.50	3.50	1.50	3.00	2.50	0.71	48.00
Safe Access Now	https://www.safeaccessnow.org	3.50	4.00	4.00	3.00	2.50	2.50	4.00	2.50	3.50	3.50	4.50	1.00	3.50	1.50	4.00	3.00	0.00	47.50
Guidance PA	https://www.guidancepa.com	4.50	4.50	3.50	3.50	2.50	2.00	3.50	2.50	3.50	5.00	3.00	1.00	2.50	2.50	3.00	3.00	0.00	47.00
WikiLeaf	https://www.wikileaf.com	5.00	4.50	2.50	2.50	3.50	1.50	4.50	3.00	4.00	4.50	3.50	1.50	2.50	1.50	2.50	3.00	0.00	47.00
Canex	https://www.canex.co.uk	5.00	5.00	3.50	2.50	2.00	2.50	3.50	3.50	4.00	4.50	2.00	1.00	2.50	1.50	3.50	3.00	0.00	46.50
National Pain Report	http://www.nationalpainreport.com	3.50	4.50	4.00	2.50	2.50	1.50	5.00	2.00	2.50	4.50	2.50	2.00	2.00	3.50	3.50	2.50	0.71	46.00
Health Europa	https://www.healtheuropa.eu	2.50	4.50	3.50	2.50	3.00	1.50	3.50	3.50	4.50	4.50	3.00	1.00	2.50	2.50	2.50	2.50	0.71	45.00
Weed News	https://www.weednews.co	4.50	4.50	3.50	1.50	2.50	1.00	3.50	1.50	4.50	4.50	2.00	1.50	2.50	1.50	3.50	2.50	0.71	42.50
Med Card Now	https://www.medcardnow.com	4.00	2.50	2.50	3.50	3.50	1.00	2.50	1.50	3.50	4.50	3.50	1.00	2.50	1.00	4.50	3.00	0.00	41.50
Weed Maps	https://www.weedmaps.com	4.00	3.50	2.00	3.50	2.00	1.50	3.50	2.00	4.50	4.50	2.50	1.50	2.50	2.00	2.00	2.50	0.71	41.50
Cannabis Clinics	https://www.cannabisclinics.ca	3.50	4.00	2.50	3.00	1.00	3.00	3.50	2.00	2.00	3.50	3.50	1.00	1.50	1.00	4.50	2.50	0.71	39.50
Doctor Oz	https://www.doctoroz.com	1.00	N/A	2.50	2.50	2.00	2.50	4.00	3.50	2.50	4.00	3.00	1.00	2.00	5.00	4.00	2.50	0.71	39.50
Ohio Marijuana Card	https://www.ohiomarijuanacard.com	3.50	1.50	3.50	2.50	2.00	2.00	3.50	1.50	3.00	5.00	2.50	1.00	3.50	1.50	3.00	2.50	0.71	39.50
Medical Cannabis	https://www.medicalcannabis.com	4.50	3.50	2.50	3.00	2.50	1.50	4.50	1.00	2.50	4.50	2.00	2.00	1.00	1.00	1.50	2.50	0.71	37.50
SepaPain	https://www.sepapain.com	3.50	2.50	2.50	1.00	1.00	1.50	3.00	1.50	2.50	4.50	1.50	1.50	2.50	5.00	3.50	2.50	0.71	37.50
People's Cali	https://www.peoplescali.com	3.50	4.00	2.50	3.00	3.00	1.00	3.00	1.50	3.50	4.50	1.50	1.00	2.50	1.50	1.50	2.00	0.00	37.50
Denver Dispensaries	https://www.denverdispensaries.net	1.50	1.50	4.00	1.50	1.00	1.50	2.50	1.50	2.50	4.00	3.00	1.00	2.50	1.50	4.00	2.50	0.71	33.50
**Total means**		3.89	3.96	3.38	3.19	2.97	2.68	3.96	2.96	3.38	4.31	3.18	1.72	2.60	3.11	3.68	3.10	0.45	48.85
**Total standard deviations**		0.89	0.94	0.78	0.98	1.24	1.21	0.77	1.08	0.72	0.51	0.83	0.74	0.69	1.60	0.93	0.62	0.35	8.13

Table 2 provides the scores for each website across each of the three sections of the DISCERN instrument. Section 1 addresses the reliability of the publication, section 2 focuses on specific details of the information about treatment choices, and section 3 consists of the overall quality rating of each website. Each item is rated based on a 5-point Likert scale, ranging from no/does not fulfill criterion (1 point) to yes/fulfills criterion (5 points)

### Trends identified across resources assessed

#### Questions 1–8: reliability of the publication

Question 1 asks if the aims of the publication are clear. Specifically, this question ascertains what the publication is about, what it is meant to cover, and who might find it useful. In general, health portals scored highest in this section, with commercial websites scoring the lowest. The mean score for this question was 3.89 (SD = 0.89), and the scores ranged from 1 to 5.

Question 2 seeks to understand if the publication has achieved its aims (the aims that were evaluated by question 1). This question is closely linked to question 1. In general, websites that scored low on question 1 also scored poorly on question 2. Health portals scored highest on this section, and commercial sites scored lowest, however, question 2 also saw lower scores for professional sites, compared to question 1. The mean score for this question was 3.96 (SD = 0.94), and the scores ranged from 1.5 to 5.

Question 3 asks if the information in the publication is relevant. This question ascertains whether the publication addresses the questions that readers might ask. It also asks whether recommendations and suggestions within the publication concerning treatment choices are realistic or appropriate. Professional and commercial websites generally scored most poorly on this section. The mean score for this question was 3.38 (SD = 0.78), and the scores ranged from 2 to 4.5

Question 4 asks whether the sources used to compile the information available in the publication are clear and accessible. Cannabis news, commercial, and professional websites generally scored very poorly on this section (the lowest scoring website was SepaPain, with a score of 1). The mean score for this question was 3.19 (SD = 0.98), and the scores ranged from 1 to 5.

Question 5 evaluates whether the dates of any source information and all publication revisions are readily available on the site. Generally health portals scored well on this question while commercial websites scored poorly. The mean score for this question was 2.97 (SD = 1.24), and the scores ranged from 1 to 5.

Question 6 asks if the source of consumer health information is balanced and unbiased. Both professional and commercial websites (and cannabis news websites to a lesser degree) scored lower in this category when compared to health portals and non-profit websites. Eleven out of 12 professional and commercial websites scored below a 3 on this item. These websites presented more persuasive and positive language when discussing cannabis, had fewer reputable or easily traceable sources, and discussed the possibility of alternatives to cannabis much less frequently as opposed to health portal or non-profit websites. The mean score for this question was 2.68 (SD = 1.21), and the scores ranged from 1 to 5.

Most websites scored comparatively higher on question 7 of the DISCERN instrument. In fact, only 4 out of 36 websites scored below a 3.5 for this question. This section asks if information presented on the website was supported by additional sources, and whether links to pages/websites with similar topic information were available within webpages. Most websites provided references and hyperlinks to other websites (such as government agencies or other pages within the website with similar topics). The mean score for this question was 3.96 (SD = 0.77), and the scores ranged from 2 to 5.

Question 8 asks if the publication refers to areas of uncertainty. For example, this question ascertains if there is discussion of the gaps in knowledge or differences in expert opinion concerning treatment choices. Commercial and professional websites generally scored very poorly on this section (i.e., Denver Dispensaries and Med Card Now). However, the lowest scoring website for this particular question was Medical Cannabis, a non-profit website. The mean score for this question was 2.96 (SD = 1.08), and the scores ranged from 1 to 4.5.

#### Questions 9–15: specific details of the information about treatment choices

Question 9 assesses whether the publication describes how the proposed treatment works. Commercial and professional websites, in general, scored most poorly on this section, as they provided little to no explanation of treatment mechanisms. Such websites typically only provided a list of treatment benefits, while providing, at most, a cursory explanation of the treatment’s physiological mechanisms. The lowest two scoring websites were Lab Blog UofM and Cannabis Clinics (both professional websites). The mean score for this question was 3.38 (SD = 0.72), and the scores ranged from 2 to 4.5.

Question 10 asks if the publication describes the benefits of each treatment. Most websites scored well on this question, with detailed descriptions of the many possible benefits associated with cannabis use included. Of the 36 included websites, 23 scored at or above a 4.5 on question 10, and only one website, Lab Blog UofM, scored below a 3.5. The mean score for this question was 4.31 (SD = 0.51), and the scores ranged from 3 to 5.

Question 11 investigates if the publication accurately and fully describes the risks of each proposed treatment. Most websites, with the exception of health portals and non-profits, scored poorly on this question. Most commercial, cannabis news, and professional websites either only discussed treatment risks briefly, or omitted mention of risks completely. The two lowest scoring websites were People’s Cali (commercial) and SepaPain (professional) with a score of 1.5 each, indicating an almost complete lack of risk warnings. The mean score for this section was 3.18 (SD = 0.83), and the scores ranged from 1.5 to 4.5.

Question 12 of the DISCERN instrument assesses whether a publication explains what would happen to a patient who did not undergo treatment. Twenty-eight websites scored a 2 or lower, with only one website scoring above a 3.5. While a variety of treatment options were often discussed with respect to pain conditions, the impact of receiving no treatment for these conditions was rarely discussed. Although some websites stated that the pain condition could be resolved without treatment, they did not directly discuss how chronic conditions could progress without treatment, or provide more details about this information. Some websites, even if briefly, supported the idea that cannabis is a preferential pain management option to opioids, claiming that it causes less damage and cannot result in addiction. The mean score for this question was 1.72 (SD = 0.74), and the scores ranged from 1 to 4.

Question 13 asks if the website takes into account the various impacts a specific treatment choice could have on an individual’s quality of life (i.e., financial strain, ability to continue work, and any potential impact on interpersonal relationships). Only two websites in this category scored higher than a 3.5, indicating an overall lack of this information. Twenty-two websites discussed this generally in terms of short-term effects of cannabis (i.e., decreased driving ability), but did not discuss long-term impacts on patients' quality of life, while the remaining 14 websites did not discuss this at all. The mean score for this question was 2.60 (SD = 0.69), and the scores ranged from 1 to 4.

Question 14 assesses whether it is made clear in the publication that there may be more than one possible treatment choice. In general, professional and commercial websites did not mention any possible alternatives to cannabis therapy for pain, and solely focused on describing cannabis benefits. In contrast, health portals scored higher for this question. The mean score for this question was 3.11 (SD = 1.60), and the scores ranged from 1 to 5.

Finally, question 15 asks if the publication provides support for shared decision-making. In other words, this question asks if the publication encourages patients to discuss treatment options with a healthcare provider, such as a physician, prior to using medical cannabis. Generally, cannabis news and commercial websites scored poorly on this section. The mean score for this question was 3.68 (SD = 0.93), and the scores ranged from 1.5 to 5.

### Recommended websites for patients and consumers

The five highest-rated websites had a mean summed score of 61.20 (out of 75), and a mean overall score (question 16) of 4.20 out of 5. All 5 websites were either characterized as a health portal or a non-profit. All of these websites scored highly on question 15, as they placed a significance on shared decision-making (i.e., discussing treatment options with friends, family, and healthcare providers). In addition, question 6 of the DISCERN instrument asks whether the publication is balanced and unbiased, and all 5 websites scored 3.5 or higher on this question as they provided more objective language, while accounting for any potential competing interests. All 5 websites aimed to provide less biased information and encouraged the reader to discuss treatment options with their family and professionals. Additional characteristics of the 5 recommended websites are provided in Table 3.

**Table 3:** Recommended websites for patients and consumers

Website name	URL	DISCERN score (sum)	Overall rating (Q16) score (mean)	Website category	Target audience	Frequency of updates
Medical News Today	https://www.medicalnewstoday.com	65.00	4.50	Health portal	Healthcare providers, researchers, patients/public	Website content is updated daily. Frequency of website update in general not available.
WebMD	https://www.webmd.com	64.50	4.50	Health portal	Patients/public	Website content is updated daily. Frequency of website update in general not available.
Very Well Health	https://www.verywellhealth.com	62.50	4.00	Health portal	Healthcare providers, patients/public	Frequency of updates is not available.
Creaky Joints	https://www.creakyjoints.org	60.50	4.00	Non-profit	Patients/public	Website updated monthly.
Arthritis	https://www.arthritis.ca	53.50	4.00	Non-profit	Patients/public	Frequency of updates is not available.

Table 3 provides additional details about the five highest scoring websites based on DISCERN instrument assessments, which may be of value to healthcare providers who seek to recommend online resources to patients about cannabis use for pain

## Discussion

The purpose of this study was to assess the quality of online cannabis consumer health information for the treatment/management of pain. We identified 36 eligible websites that contained cannabis consumer health information for pain. The mean summed DISCERN score was 48.85 (SD = 8.13, range from 33.50 to 65.00). The mean overall score (question 16) was 3.10 (SD = 0.62, range from 2.00 to 4.50). In general, health portal and non-profit websites comprised the top 40% of the overall DISCERN ratings (question 16), while the remaining 60% consisted of news, professional, and commercial websites.

It is hoped that the present study’s findings will aid healthcare professionals in their understanding of the quality of information surrounding the intersection of cannabis and pain available to patients and the general public online. Published research that has assessed the quality of online patient information in general has indicated that many commonly visited websites are maintained by individuals or organizations with direct financial interests in promoting health treatments or therapies (Kunst and Khan 2002). Often, the information provided on these websites have been found to be incomplete, anecdotal, or not representative of evidence-based research, and tended to over-exaggerate positive aspects and underplay (or completely omit) information surrounding risks and negative side effects (Macedo et al. 2020; Chen et al. 2018). Additionally, it has been found that 60% of internet users seeking medical information believed that what they obtained online was “the same” or “better” than the resources obtained from their physician (Diaz et al. 2002). Further to this, it has been found that while patients are largely able to discern biases in information provided on commercial websites, they often also reject high quality websites solely based on website design (Sillence et al. 2007). Another study also identified that source credibility had no significant effect on a consumer’s evaluation of the quality of online information (Bates et al. 2006).

### Comparative literature

It is reasonable to infer that patients who seek cannabis information online are not exempt from the behaviors found in the aforementioned studies, which may put them at risk of experiencing negative health outcomes. One study characterized the interest in using cannabis as a treatment for cancer online, and the propagation of this information on social media. The authors found that false news stories and advertisements on social media which claimed that cannabis can cure cancer garnered more online interactions than those debunking these claims (Shi et al. 2019). Furthermore, another study identified that many websites promote the view that cannabis use by pregnant women and children is entirely safe (Keyhani et al. 2018). Perhaps most worrying, another study also found that online cannabis misinformation is responsible for lowering the risk perceptions among adolescents, thereby potentially lowering their inhibitions, leading them to engage in use (Belenko et al. 2009). With respect to pain-specific information, there have also been a few studies assessing the quality of online consumer health information in the context of complementary or alternative medicine, which is comprised of a diverse group of therapies of which cannabis is sometimes included. One study evaluated the quality of complementary or alternative medicine consumer health information for arthritis, and found that many websites lacked source transparency and risk reporting (Ng et al. 2021a). Two other studies investigated the quality of complementary or alternative medicine consumer health information for low back pain and neck pain, respectively, and reported that many websites did not adequately report the risks or adverse side-effects of treatment options adequately (Ng and Gilotra 2020; Ng et al. 2021b). Similar findings have also been reported with respect to commonly used herbal products such as St. John’s wort (Thakor et al. 2011), kratom (Ng et al. 2021c), and ephedra (Ng et al. 2021d). Another issue of concern includes the fact that across numerous jurisdictions, even where medical cannabis is legalized, physicians report lacking knowledge and information, while acknowledging their need for greater and continuing education, on this topic (Kansagara et al. 2020; Philpot et al. 2019; Ziemianski et al. 2015; Ng et al. 2021e; Zolotov et al. 2018). Collectively, it is clear that cannabis misinformation is commonly found on the internet, and the present study’s findings only reinforce the need for healthcare professionals to be actively aware of this information quality in order to better assist their patients in identifying trustworthy and accurate cannabis resources online.

### Strengths and limitations

A notable strength of our study includes the use of the DISCERN instrument to assess the quality of our subset of websites, as it has been found to be both valid and reliable in assessing the quality of consumer health information. By interpreting the DISCERN instrument scores across websites, and using a series of search strategies that replicate typical patient behavior, it is likely that our findings are generally also applicable to other websites that discuss cannabis in the context of pain. This helps to provide insight into the type and depth of counseling which should be afforded to patients seeking information about cannabis for pain online. Furthermore, website screening, data extraction, and quality assessments were all performed independently and in duplicate. All three authors then met to discuss any discrepancies without unduly modifying original scores.

With respect to limitations, it should be acknowledged that all websites were assessed cross-sectionally. The internet is constantly changing, and the content on this subset of websites are likely no exception. An additional limitation includes the fact that only English-language websites were included and assessed, based on study resource limitations. We must also acknowledge that different search results may have appeared despite identical Google search strategies, if conducted in different regions’ native languages (i.e., Dutch for Google.nl, Spanish for Google.com) or geographic locations (i.e., conducting the search in the Netherlands or the USA), despite searching using incognito mode on the Google Chrome browser. It may be of value to assess the quality of information provided on websites originating from other countries (i.e., Mexico (Secretaría de Gobernación 2017) or South Africa (South Africa Health Products Regulatory Authority 2017), which both decriminalized cannabis for medical use in 2017), especially as a global trend tends towards legalization across more jurisdictions around the world.

## Conclusion

Given the fact that a high proportion of individuals suffer from pain globally, a large subset of this population undoubtedly seeks consumer health information online about cannabis. The purpose of this study was to assess the quality of online cannabis consumer health information for the treatment/management of pain. Our findings indicate that the consumer health information available at the intersection of cannabis and pain is commonly incomplete and biased. While health portal and non-profit websites generally provide higher-quality information, commercial, professional, and cannabis-focussed news websites tended to only present the positive aspects of cannabis while downplaying the potential risks of use. Our results also corroborates findings from a number of published studies which have reported that consumers may be at risk of making poor health-related decisions following information-seeking online, both in general and in the context of cannabis use. Healthcare providers need to be aware of the information their patients seek pertaining to cannabis online and should be prepared to guide them in identifying high-quality resources which promote the safe and effective use of this therapy.

## Data Availability

All relevant data are included in this manuscript.
